# An enhanced *C. elegans* based platform for toxicity assessment

**DOI:** 10.1038/s41598-017-10454-3

**Published:** 2017-08-29

**Authors:** Huajiang Xiong, Catherine Pears, Alison Woollard

**Affiliations:** Department of Biochemistry, University of Oxford, South Parks Road, Oxford, OX1 3QU UK

## Abstract

There is a well-defined regulatory framework governing the approval of chemicals for use as pharmaceuticals or release into the environment. Toxicity assessment is thus a major hurdle in the compound discovery pipeline, currently involving large scale animal testing. The search for alternative testing platforms is therefore an important priority. We have developed a convenient, low cost assay utilising the nematode *Caenorhabditis elegans*, to rapidly assess both acute toxicity and developmental and reproductive toxicity (DART). However the worm is protected by a robust cuticle that forms a barrier to chemical uptake. We assessed mutants with altered cuticle properties to identify sensitized strains optimized for toxicity assays. Evaluating the trade-off between increased permeability and reduced fitness identifies *bus-5(br19)* as the most suitable strain for chemical exposure. We demonstrate the applicability of this assay for a range of chemicals with differing properties, including a modified exposure protocol for volatile or less soluble compounds. This work enhances the effectiveness of *C. elegans* for convenient toxicity assessment, which could contribute to a reduction in the use of vertebrates particularly at the crucial early stages of product development. Strains identified in this work will also enhance the sensitivity of *C. elegans* based drug discovery platforms.

## Introduction

There are stringent requirements for toxicity testing of chemicals involving both acute toxicity and developmental and reproductive toxicity (DART), particularly pertinent to the petrochemical, agrochemical and cosmetics industries in which chemicals are released into the environment. This testing involves large numbers of animals. Currently the regulatory framework for DART testing demands one (OECD 414) and two (OECD 416) generational studies of rats and rabbits and account for the use of thousands of animals per year^1^. Home Office figures reveal 556,000 animals were used in regulatory tests in 2015 in the UK alone (https://www.gov.uk/government/statistics/statistics-of-scientific-procedures-on-living-animals-great-britain-2015). Although the pharmaceutical industry is subject to different regulations by the Medicines and Health Care Products Regulatory Agency in the UK, any drugs taken chronically or by children have potential for DART and similar testing requirements apply.

The financial and ethical costs of using such large numbers of animals, together with the time taken for testing (resulting in opportunity costs for product development), means that alternative testing platforms are highly desirable^2^. Indeed animal testing has been banned within the European Union for cosmetics further driving the need for alternatives. Such platforms are envisioned to give an indication of toxicity potential early in the product development pipeline in order to eliminate high-risk compounds without the need to test on vertebrates. Initiatives to reduce animal testing in DART have focused mainly on outcomes from cell-based assays and the use of *in silico* approaches, yet none of these have been able to incorporate the complexity of the reproductive and developmental life cycle.

The nematode *Caenorhabditis elegans*, with its ease of manipulation and storage, short life cycle and low husbandry costs, has long been recognised as one of the premier model organisms for biological research. The worm shares many fundamental biological processes with humans, has a completely sequenced and fully annotated genome (together with a wealth of genomic reagents and resources), and is highly tractable at both the genetic and biochemical level. Comparative genomic studies have revealed that *C. elegans* contains many genes homologous to mammals^3, 4^ which make the worm a powerful biomedical tool for disease modelling, drug discovery and toxicity assessments^5–9^. The transparent nature of the worm, coupled with the invariant and completely described cell lineage, means that development is incredibly well defined, at single cell resolution, with a great many well described phenotypes associated with particular developmental perturbations. Development involves the vast majority of molecular signalling pathways common to human development and cell behaviour, and thus *C. elegans* offers great potential for developmental toxicity testing. In addition, the hermaphrodite nature of *C. elegans*, coupled with the short (3 day) generation time, means that trans-generational studies are particularly convenient, offering advantages for the study of reproductive toxicology.

*C. elegans* has been used to identify pharmacological targets for various human diseases, and is gaining increasing attention as a promising multicellular alternative for toxicity testing^10–13^. Positive predictive power of *C. elegans* for toxicological research has been shown. For example, one study demonstrated that 89% of compounds compromising egg viability in the worm also have known developmental effects in mammals^14^, while a study of 47 compounds associated with mammalian reproductive toxicity showed up to 69% concordance between *C.elegans* data and ToxRefDB mammalian data^12^. In a more extensive study, toxic effects associated with exposure of *C. elegans* to over 900 chemicals were compared with ToxCast data from zebrafish, rats and rabbits^15^. The authors found concordance of *C. elegans* data with data from rats and rabbits of between 45 and 53% across a range of doses, which is only slightly lower than the concordance between rat and rabbit data (58%).

Previous testing regimes in *C. elegans* have tended to focus on rather specific endpoints, such as increased apoptosis^12^, or have reported on only developmental but not reproductive effects^14, 16^, or reproduction only^17^ and this has been proposed to lead to a high percentage of compounds missed in these assays. For example, in an egg viability assay 75% of compounds which did not reduce egg viability do cause toxic effects in mammals^14^. In this work, we establish a rapid, convenient yet quantifiable assay capturing both acute toxicity and DART, by covering the whole developmental and reproductive life cycle.

One potential disadvantage of using nematodes in a chemical testing regime is that they are protected by a robust cuticle made of complex layers of collagen that functions as a physical barrier against impact of chemicals, toxins and pathogens^18–22^. One study revealed the concentration of 5-hydoxytryptophan to be 100–1000-fold lower inside an exposed worm than the concentration in the medium^17^. A high throughput screen of over 1000 small molecules revealed problems with drug accumulation inside worms in over 90% of cases^23^. This poses a problem for chemical testing because uptake appears to be compromised in *C.elegans*, and thereby toxic effects underestimated^18^. Mutant strains bearing compromised cuticles have been shown in some instances to facilitate chemical uptake, leading to increased sensitivity to particular agents.

Here, we have selected a group of candidates carrying mutations in the genes *agmo-1*, *bus-5*, *bus-8*, *bus-16* and *bus-17*, all of which are known for their compromised cuticle. The Bus (bacterially unswollen) mutants were originally identified by their resistance to the nematode pathogen *Microbacterium nematophilum* ^24, 25^. *bus-8* encodes a predicted glycosyltransferase that is required for epidermal integrity and production of the cuticle surface; mutants have been shown to display enhanced sensitivity to the neurotoxins nicotine, 1-phenoxypropan-2-ol and ivermectin^26^. *bus-5* encodes the dTDP-glucose 4,6-dehydratase *rml-2* and is required for dTDP rhamnose biosynthesis and is suggested to be a cuticular component^27^. *bus-17* encodes a predicted galactosyl-transferase required for cuticle integrity, as evidenced by bleach sensitivity^24, 28^. *bus-16* is uncloned but is also required for proper cuticle integrity^24^. *agmo-1* (encoding an alkyl glycerol mono-oxygenase) is required for epidermal lipid metabolism involved in cuticle integrity^29^.

We determined cuticle permeability for the five selected strains and assessed this against potentially reduced fitness, which would be undesirable in a testing strain. Optimised strains were tested against a range of chemicals and the *bus-5* mutant was found to combine features of enhanced permeability and chemical sensitivity with negligible fitness consequences. Overall, we have established an enhanced assay for both acute toxicity and DART assessment in *C. elegans* and an optimised strain for testing.

## Results

### Establishment of a plate based assay for toxicity assessment

**Figure 1:**
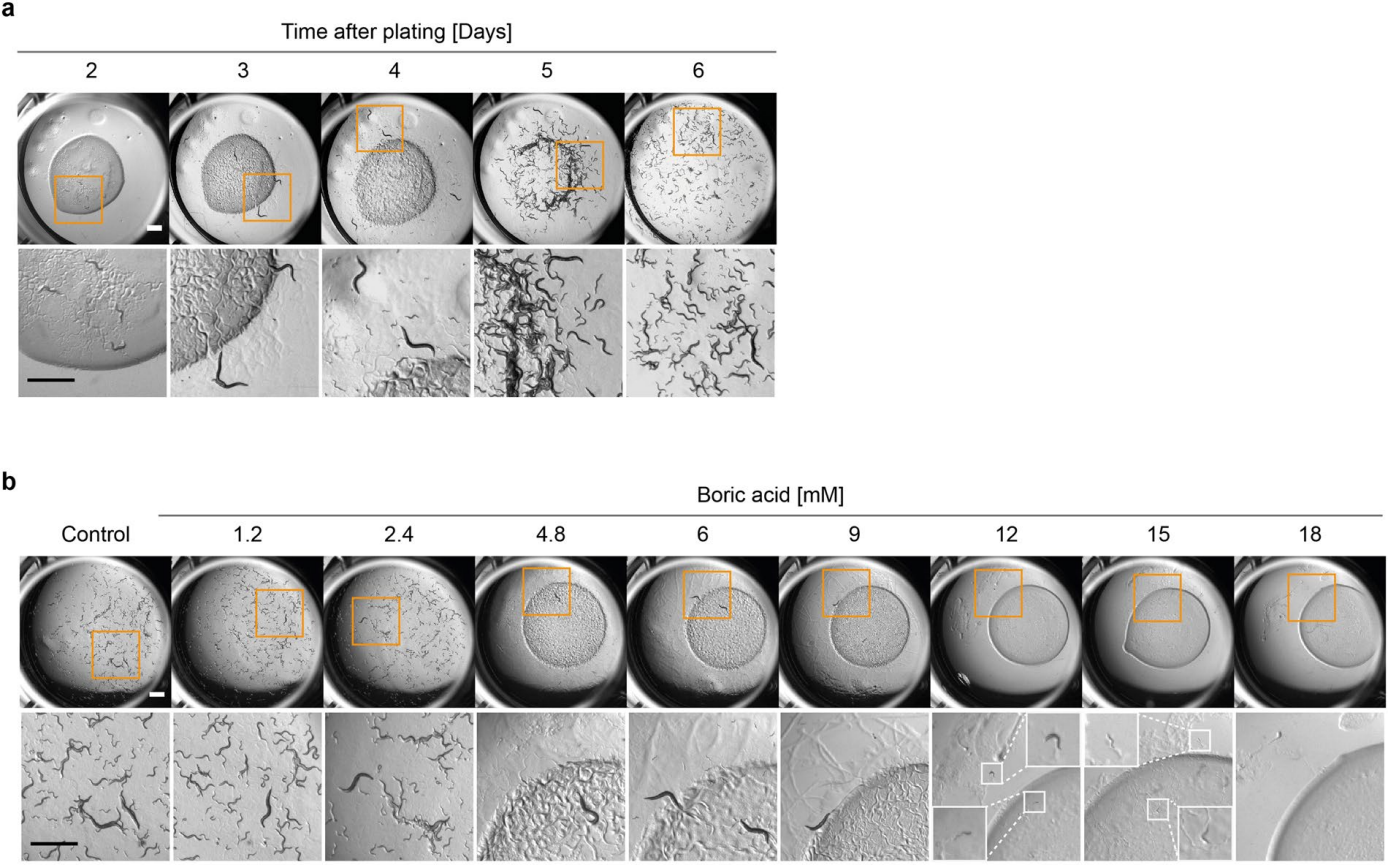
Convenient plate assay for toxicity assessment in *C. elegans*. (**a**) Newly hatched WT (N2) larval worms (5 individual L1 animals) were plated onto NGM agar seeded with bacterial food in a 24-well plate format and allowed to grow and develop. Individual wells were assessed and photographed daily for 6 days. (**b**) Larval worms were plated as in a in the presence or absence of different concentrations of boric acid. Wells were imaged on day 6, when control animals plated in the absence of chemical had depleted the bacterial food source. Orange boxes indicate the magnified area shown in the panels below. Small white boxes indicate the area magnified within the larger insets. Scale bars are 1 mm.

Worms can easily be grown in a 24-well plate format on solid media throughout their developmental and reproductive cycle and this has previously been used as a convenient toxicity screening platform^30^. Firstly, we established that the optimal number of worms that can be grown on an individual well without exhausting the food supply before the completion of the reproductive cycle (one generation) was five animals (data not shown). When five L1s were picked onto a fresh well containing a lawn of OP50 bacteria, it was possible to observe both the development of the five P0 animals as well as the production and development of the next generation (F1). Wells were photographed over a period of 6 days, until the bacterial food source became completely exhausted and animals began to starve (Fig. 1a). The original larvae developed into adult worms by day 3 and significant numbers of live progeny are apparent by day 4. As food becomes depleted, the worms first accumulate at the edge of the food circle (a behaviour known as bordering) where bacterial growth is thickest, and this is apparent at day 5, before the bacterial lawn completely disappears (day 6). Developmental and reproductive rates were highly reproducible between replicate wells (data not shown).

In order to determine whether this fitness assay would be suitable for testing chemical toxicity we exposed the worms to different concentrations of boric acid, a widely used antiseptic that is known to cause DART in mammals (Fig. 1b). High concentrations of boric acid (12 mM and above) caused acute toxicity (death of P0 animals). Exposure to concentrations between 4.8 mM and 9 mM caused increasing developmental delay with associated delay in food depletion, with the higher concentration (9 mM) inhibiting progeny production (Fig. 1b). Exposure to concentrations of 12 mM or greater led to the death of the P0 animals. Thus, this assay can distinguish acute toxicity from DART and is simple and rapid to perform on solid medium (the preferred substrate for *C. elegans* analysis). The advantage of the assay is that it allows assessment of the same population of animals over the time course of chemical exposure. A potential disadvantage is the relatively high concentrations of chemical required to see an effect, with associated implications for cost and comparison with mammalian data.

### Identification of testing strains with enhanced permeability

**Figure 2:**
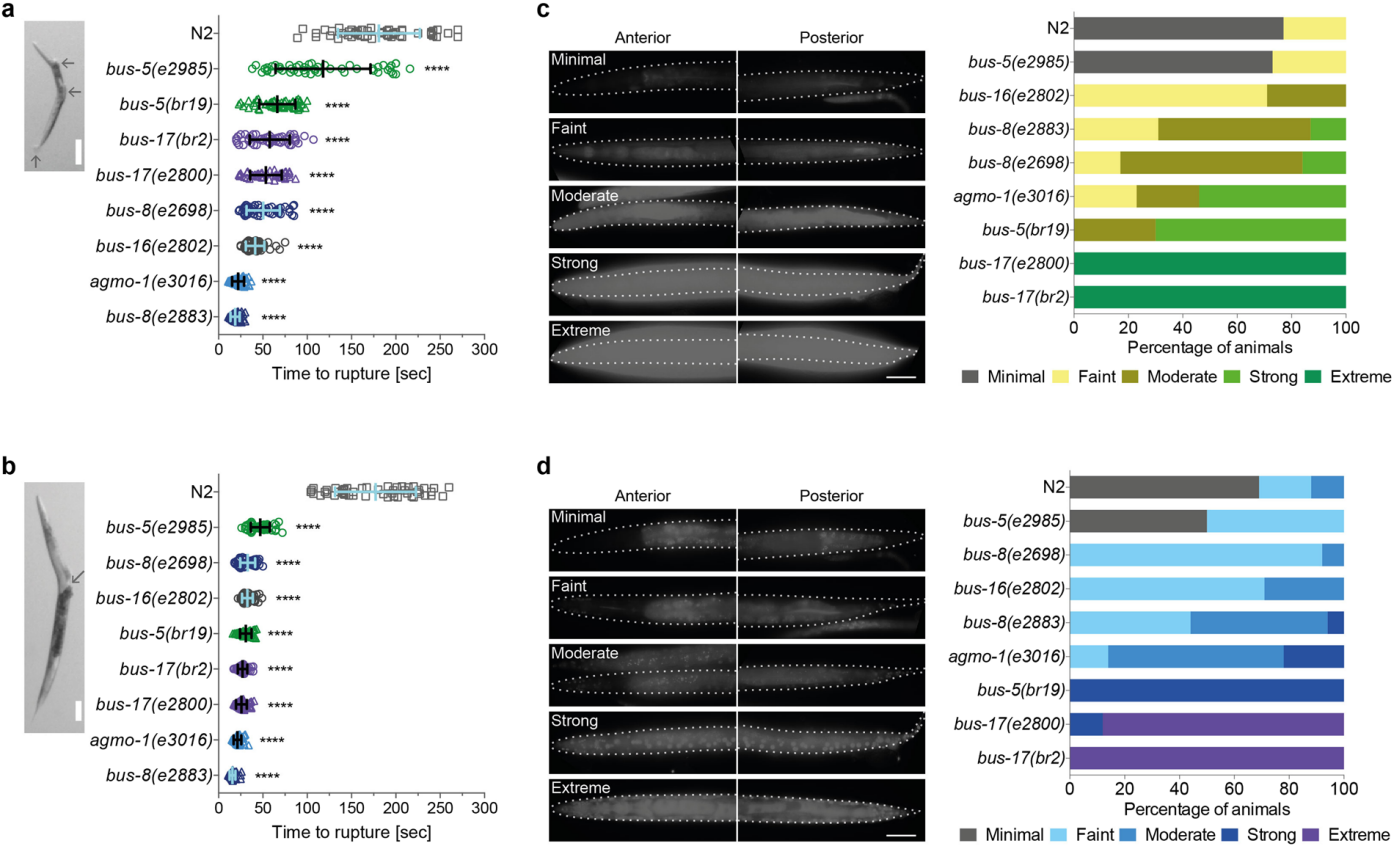
Assessment of permeability of strains with compromised surface properties. (**a**) Larval or (**b**) adult worms were exposed to sodium hypochlorite (2%v/v) and the time taken for the animal to rupture measured (as shown in inset boxes, where arrows indicate rupture site). Time taken to rupture for individual worms was plotted for each strain, as indicated (n = 50). Mean ± standard deviation (SD) is shown. All the mutant strains tested displayed significantly faster rupture time than the wild type N2 strain (p < 0.0001). L4 animals were exposed to the fluorescent dyes (**c**) acridine orange (AO) or (**d**) DAPI to monitor permeability of the worm. Fluorescence intensity was categorized in live stained animals into 5 classes as shown. Dotted lines mark the body outline of the worms. Quantification of the proportion of live L4 animals in each class is shown in the (n≈10). Scale bars represent 100 μm (**a** and **b**) or 50 μm (**c** and **d**).

The relative insensitivity of *C. elegans* to chemicals is thought to be caused by the tough exterior cuticle imposing a barrier to uptake. We hypothesized that this could be overcome by using mutants with compromised cuticle integrity. To test this we assembled a panel of mutants with various defects in cuticle structure and assessed their permeability. Previous reports have utilised assays based on the time taken for the cuticle to rupture upon exposure to bleach (commonly used in *C. elegans* research to dissolve mothers and liberate eggs), and relative permeability to fluorescent dyes^19, 24, 26, 29, 31–36^. Comparative bleach sensitivity data are shown in Fig. 2a and b. All the mutants tested displayed more rapid rupture at both larval and adult stages, indicating weakened cuticular integrity. Next, we tested uptake of two fluorescent dyes, acridine orange (AO) and 4′,6-diamidino-2-phenylindole (DAPI), in live L4 animals. The intensity of fluorescent dye accumulation was scored (Fig. 2c,d). All mutants showed increased dye permeability compared with the wild type (WT) control strain N2, with similar patterns of uptake observed for the two dyes. Where two alleles of the same gene were tested, the most permeable was selected for further analysis (*bus-5(br19), bus-8(e2883)* and *bus-17(br2)*).

### Assessment of potential fitness trade-offs in cuticle mutants

**Figure 3:**
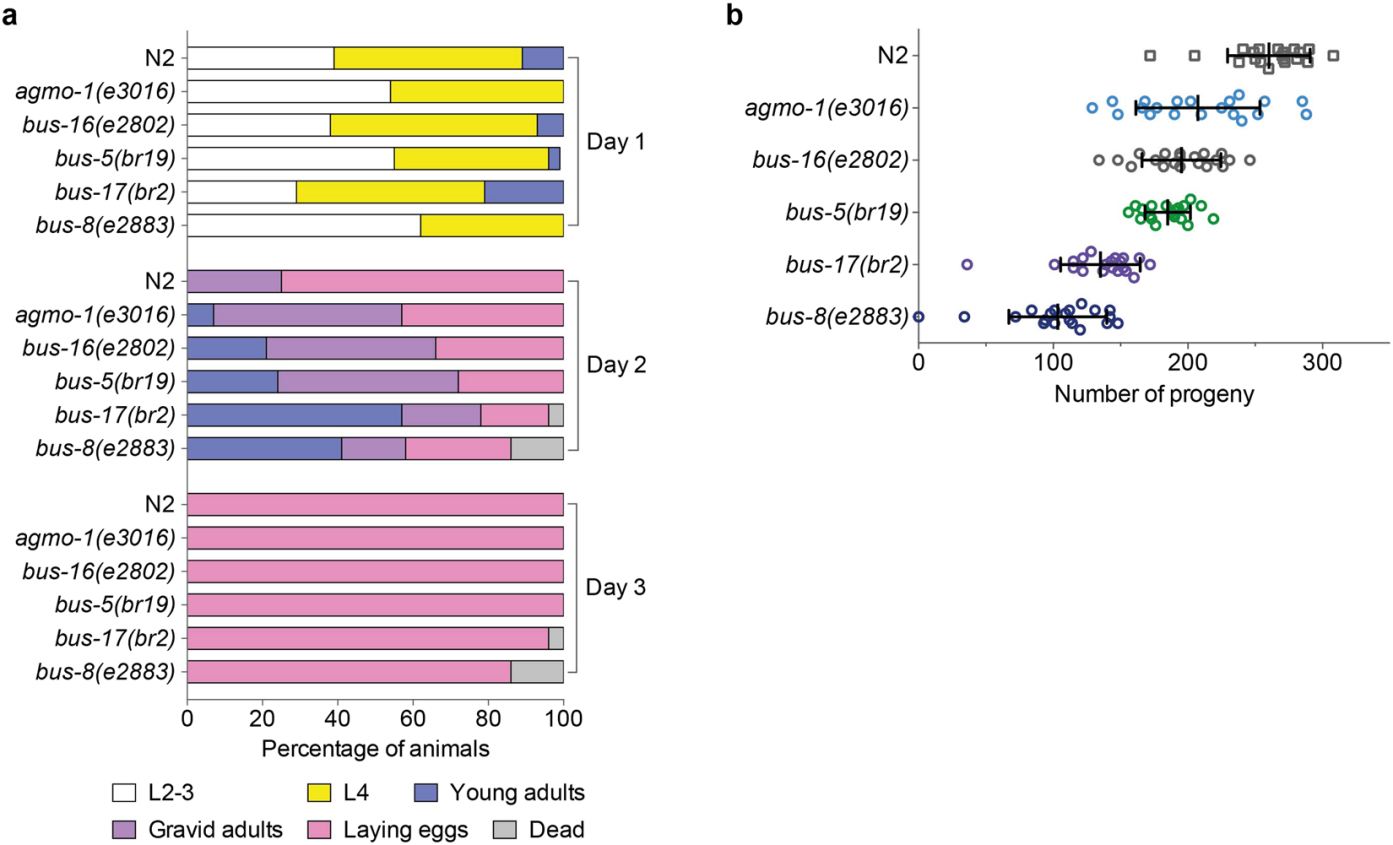
Fitness comparison of candidate testing strains. (**a**) Developmental speed was monitored by plating L1 animals on day 0 and assessing developmental stage over the subsequent 3 days (L2-L3, L4, adult, adult containing eggs (gravid) and adults laying eggs), n = 28 for each strain. (**b**) Total brood size per hermaphrodite animal of indicated strain, n = 20. Mean ± SD are shown. All mutant strains displayed a significantly reduced brood size compared with WT (p < 0.0001). *agmo-1, bus-16* and *bus-5* strains were not significantly different from one another, however both *bus-17* and *bus-8* mutants displayed a significantly reduced brood size compared with the first three mutants (p < 0.0001).

It is possible that permeable mutants with compromised cuticle features would display phenotypic defects that would render them unattractive for toxicity testing. Therefore we investigated developmental speed and brood size in our selected alleles (Fig. 3). 75% of WT animals were laying eggs by day 2, whereas all the mutants tested exhibited some degree of delay, with *bus-17* and *bus-8* showing the greatest delay, coupled with the early death of a proportion of animals (Fig. 3a). In terms of brood size measurement (i.e. the total number of progeny per worm), all the mutants produced a significantly smaller brood (Fig. 3b), with *bus-17* and *bus-8* again showing the most dramatic decrease, significantly lower than the other three mutants. Based on these results, we concluded that *bus-17* and *bus-8* mutants were most compromised in terms of overall fitness and they were therefore excluded from subsequent analyses.

### Chemical sensitivity in selected strains

To demonstrate the benefit of the sensitized strains for developmental and reproductive toxicity assays, we exposed L1 larvae to a small collection of chemicals with known toxicity effects on mammals (Table 1). Chemicals were dissolved in water, DMSO or a DMSO/isopropanol mix depending on solubility (see table legend). The maximum concentration of these solvents that did not show developmental toxicity was determined for wild type N2 and the selected mutant strains (Supplementary Figure S1), with the mutant strains not showing enhanced sensitivity. The three selected strains, *agmo-1*, *bus-5* and *bus-16* all showed enhanced sensitivity to a range of different chemicals (Table 1), confirming our hypothesis that the cuticle represents a significant impediment to chemical uptake. Some differences between the strains were observed, however, with *bus-16* being least sensitive to most chemicals and *bus-5* being the most sensitive.

**Table 1:** Chemical toxicity assessment in selected sensitised strains.

Application	Chemical	Conc	N2		agmo-1(e3016)		bus-5(br19)		bus-16(e2802)	
			DART	Acute	DART	Acute	DART	Acute	DART	Acute
Pharmaceuticals	Boric acid [mM]	0.6					+			
		6	+		+			+	+	
	Warfarin [μM]	0.6					+			
		6					+			
		30			+		+			
Agrochemicals	Aldicarb [nM]	0.8								
		8	+			+		+	+	
	Fenoxycarb [nM]	0.5			+		+			
		5	+			+		+	+	
	Spirotetramat [nM]	2	+		+		+		+	
		4		+		+		+		+
Petrochemical	Piperazine [mM]	0.6			+		+		+	
		1.5	+		+		+		+	
		6	+		+			+	+	

Toxicity assays were set up in 24-well plates as described in the legend to Fig. 1 using a broad range of chemicals. Effects were scored as either delayed development (DART) or acute toxicity (death of P0 animals before laying any eggs) on the day that food was cleared from control wells (containing untreated animals of each strain). ^+^Indicates an observable effect. All chemicals selected for testing have been reported to show both DART and acute toxicity in mammals according to the ToxCast database (https://www.epa.gov/chemical-research/toxcast-dashboard), PubChem database (https://pubchem.ncbi.nlm.nih.gov) or Pesticide database (http://www.pesticideinfo.org). Chemicals were dissolved in water (boric acid), DMSO (warfarin, piperazine), DMSO/isopropanol (aldicarb, fenoxycarb, spirotetramat).

### Quantification of toxicological effects

**Figure 4:**
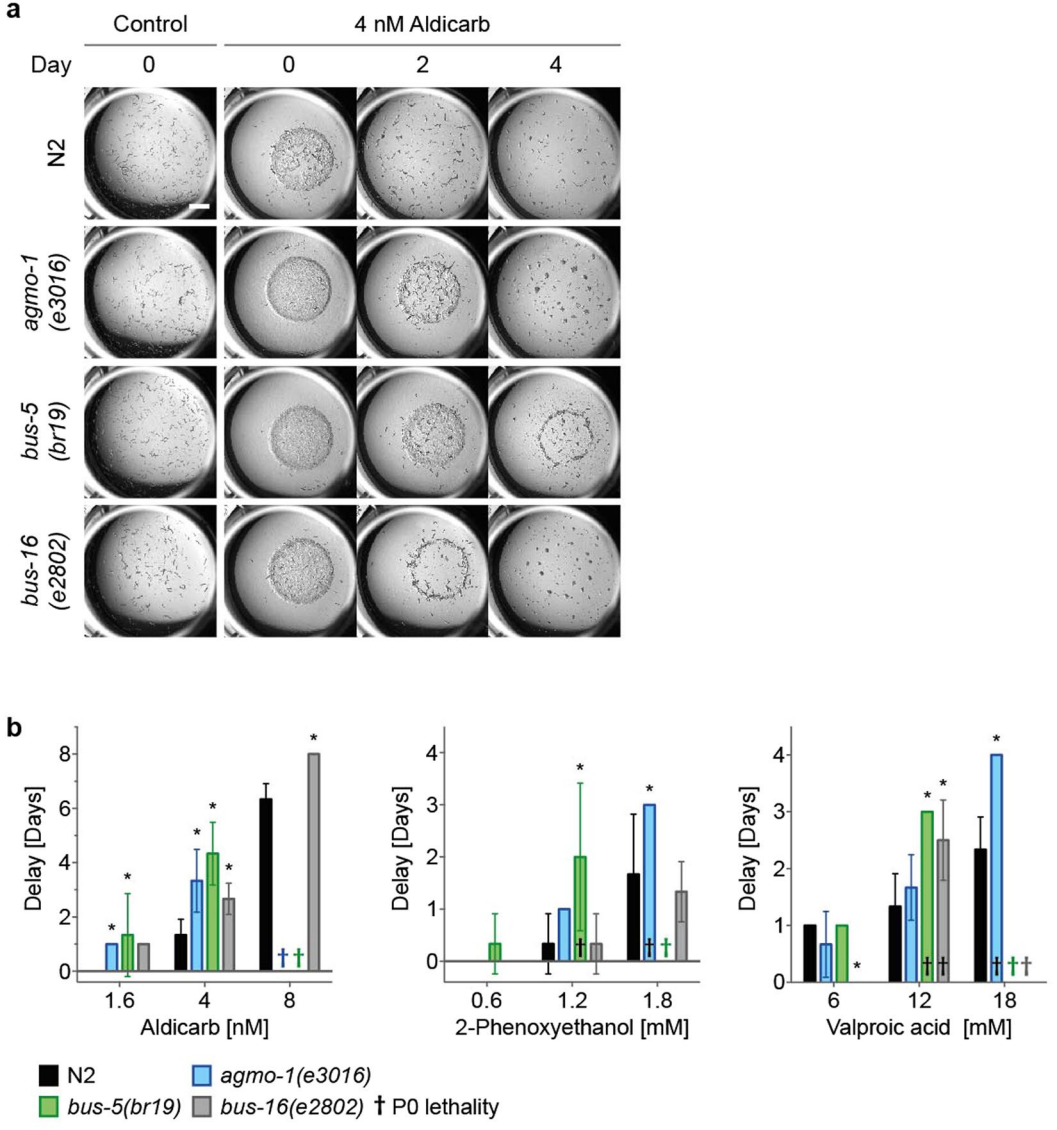
Quantitative assessment of DART in sensitised strains. Toxicity assays were set up in 24-well plates as described in the legend to Fig. 1, in the presence of different concentrations of either aldicarb, 2-phenoxyethanol or valproic acid. Wells were scored daily for food depletion and the day that control worms without chemical depleted the food source was defined as day 0. (**a)** Photographs of representative wells for WT, *agmo-1*, *bus-5* and *bus-16* control worms in the absence of chemical at day 0 and in the presence of 4 nM aldicarb at days 0, 2 and 4. Scale bars are 1 mm. (**b**) Any delay relative to the control was plotted over a range of concentrations. ^†^Indicates lethality of plated animals within timeframe of assay. Bars represent the mean of three replicates ± SD. *p < 0.01 compared to the WT control strain under the same conditions. Aldicarb was dissolved in DMSO/isopropanol, 2-phenoxyethanol in DMSO and valproic acid in water.

Although this assay was successful in distinguishing acute toxicity from DART, it became apparent that different degrees of developmental delay could be observed in the different chemical exposures (and in the different strains), and that a non-subjective way of scoring these was required. The most visually striking event in our plate assay relates to consumption of the bacterial food by the worms. The time at which the bacterial lawn is completely cleared thus provides an objective, numerical readout of population size and health (i.e. developmental and reproductive rate). The day at which the strain grown in the absence of chemical completely depleted the food was set at zero, and delays induced by chemical exposure were noted, up to 10 days if necessary, thus providing a high degree of granularity to the assay. If chemical exposure led to either acute toxicity (death of P0 animals) or lack of viable progeny (no F1 animals), these were noted separately, as the bacterial lawn would never be depleted under these conditions. We validated this approach on a small range of compounds, including aldicarb (previously tested in Table 1) and two other compounds associated with DART, 2-phenoxyethanol and valproic acid (VPA). As a representative example, Fig. 4a shows photographs of the developmental delay in the presence of 4 nM aldicarb. At day 0 (i.e the day the control wells, containing strains not exposed to aldicarb, were cleared) some delay in food depletion was apparent in all strains including WT exposed to 4 nM aldicarb, with *agmo-1* and *bus-5* showing the fewest worms (and therefore the least food consumption). Analysis on subsequent days revealed that the delay is exacerbated in all three mutant strains, with *bus-5* showing the greatest delay as bordering is still apparent on day 4 when all others had cleared the food. If this was scored as in Table 1, all three strains would have been scored the same, thus demonstrating the enhanced discriminatory power of the food depletion assay.

When this assay was performed using all three chemicals (Fig. 4b) enhanced sensitivity of mutant strains was confirmed. Thus, this assay can be used for distinct chemicals using different solvents (Fig. 4b) detecting both different degrees of DART and acute toxicity. For example, 8 nM aldicarb caused acute toxicity of *bus-5* and *agmo-1* worms, compared to DART in WT (N2) and *bus-16* animals, whereas 18 mM VPA caused acute toxicity in *bus-5* and *bus-16* and DART in WT and *agmo-1*. *bus-5* displayed acute toxicity at the highest concentration of all three chemicals tested and the highest level of DART following exposure to lower concentrations.

### Alternative exposure protocol for volatile or insoluble compounds

**Figure 5:**
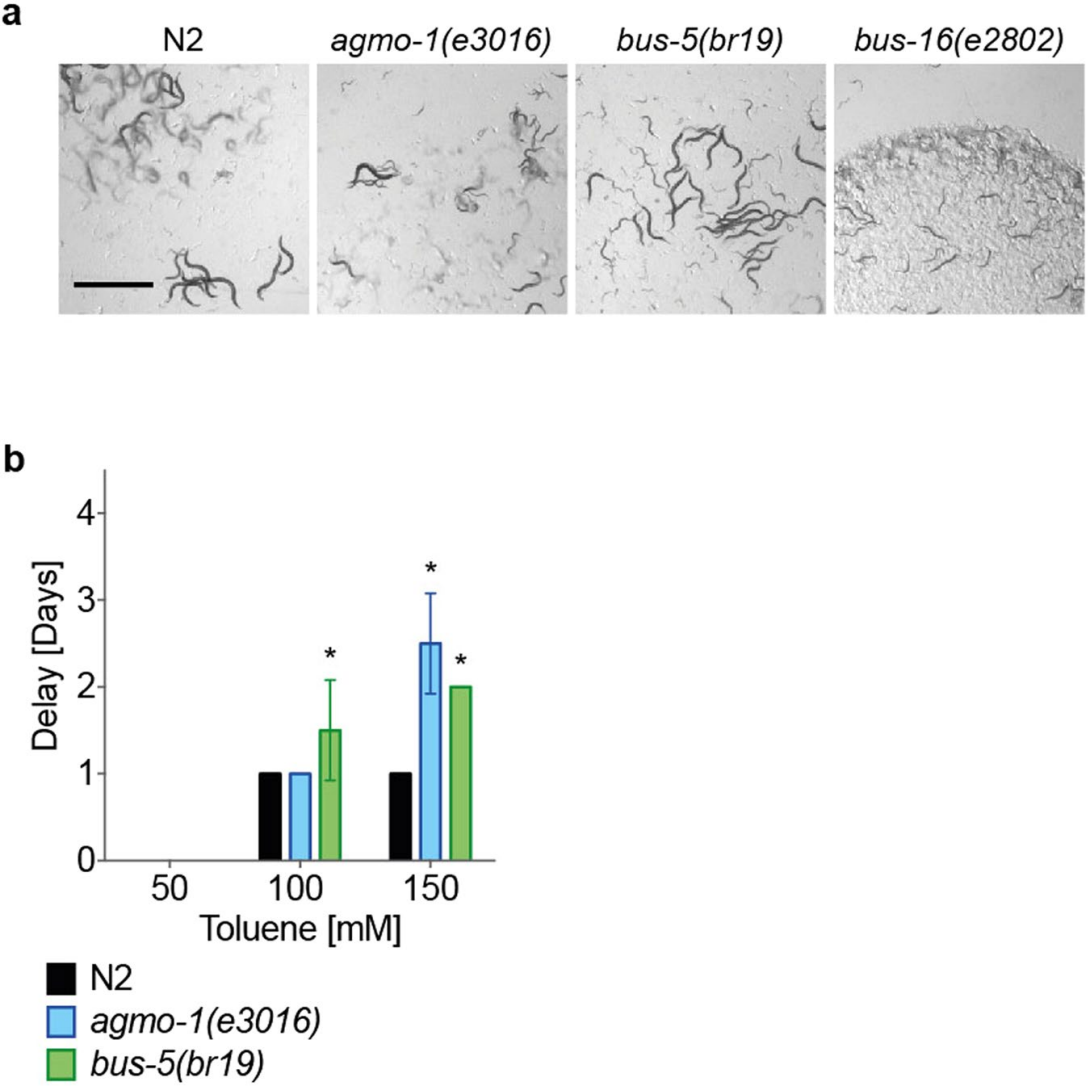
Modification of toxicity assays for volatile compounds. (**a**) WT, *agmo-1*, *bus-5* and *bus-16* L1 larvae were exposed in the 24-well plate assay under sunflower oil and photographed on the day the WT N2 worms had depleted the food source (day 0). Scale bars are 1 mm. (**b**) Dissolving toluene in sunflower oil facilitated exposure at higher concentrations up to150mM in the quantitative, food-depletion assay as in Fig. 4. Bars represent the mean of four replicates ± SD. *p < 0.01 compared to the WT control strain under the same conditions.

Next, we considered how our exposure protocol could be adapted for use with volatile compounds that would be subject to evaporation from the surface of the wells. In addition, some compounds, such as toluene, showed no effect at the maximum concentration soluble in 1% DMSO (3 mM, data not shown). Vegetable oil has previously been used as an alternative solvent for exposure of worms to chemicals^37^. Chemical exposure is achieved by overlaying the entire surface of the well with oil containing the compound. The worms live at the interface between the oil and the solid media while being exposed to compound. We tested whether sunflower oil would affect development of the mutant strains relative to wild type and found that *bus-16* displayed a developmental delay relative to WT when grown in the presence of oil (Fig. 5a) and so it was excluded from further analysis. Using this solvent allowed us to increase the concentration of toluene in chemical exposure studies to levels that revealed developmental effects (Fig. 5b). Both *agmo-1* and *bus-5* mutants showed enhanced sensitivity to toluene under these conditions.

### Selection and validation of optimised testing strain

**Figure 6:**
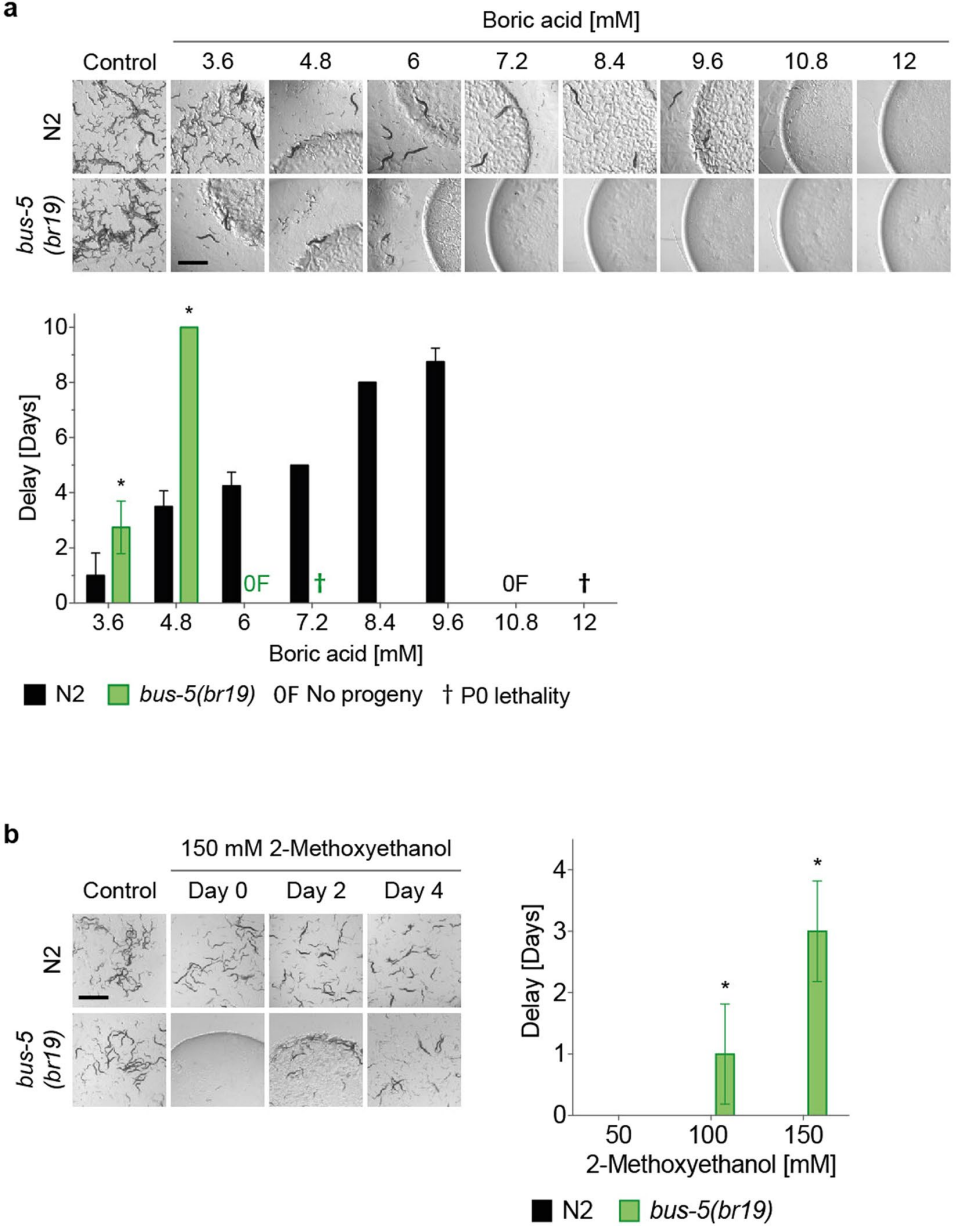
Comparison of sensitivity of wild type and *bus-5(br19)* to boric acid. Toxicity assays were set up in 24-well plates as described in the legend to Fig. 1 using wild type and *bus-5* L1 larvae exposed to a range of concentrations of (**a**) boric acid in water and (**b)** 2-Methoxyethanol in sunflower oil. Images were taken when control animals not exposed to chemical had depleted the food. Scale bars are 1mm. Quantitative analysis of delay in food depletion is depicted in the bar charts adjacent to the images. 0F1: absence of progeny; ^†^P0, lethality of plated animals within timeframe of assay. Bars represent the mean of four replicates ± SD. *p < 0.01 compared to the WT control strain under the same conditions.

Taking into account all of the data, we concluded that *bus-5* offers the optimal combination of chemical sensitivity and fitness. To validate this selection we compared the effect of a range of boric acid concentrations on *bus-5* and wild type N2 worms (Fig. 6a). *bus-5* animals showed increased sensitivity across a range of concentrations of boric acid, for example at 4.8 mM causing an enhanced delay to food depletion of 6 days compared to wild type. Finally, we assessed 2-methoxyethanol using the oil-based assay (Fig. 6b), as we had been unable to detect an effect on any strains in our standard plate assay. DART was only observed with *bus*-5, and not WT, revealing an effect that would otherwise have been missed.

## Discussion

In this work we have established an enhanced, easily quantifiable *C. elegans*-based toxicity assay that has a number of advantages over currently available platforms, including cost, rapidity and the applicability associated with analysis of a whole organism throughout its developmental and reproductive life cycle. The assay provides robust data using very small amounts of chemicals in the 24-well plate format and can be easily established without the use of specialist equipment.

Importantly the assay can be exploited to reduce the number of vertebrate animals used in toxicity studies. The strength of the assay is its ability to identify high-risk compounds early in the discovery pipeline, when only small amounts may be available, and eliminate them before testing on animals. Similarly, the relatively high throughput offered by this *C. elegans* testing platform will facilitate medicinal chemistry approaches where promising lead compounds are associated with high toxicity risk. In this situation small quantities of a large number of related compounds can be tested to avoid key alerts.

In contrast to previously reported assays using *C. elegans* ^13, 14, 16, 17^, this platform integrates a variety of toxicological endpoints including acute toxicity, developmental delay and reproductive defects, thus offering a versatile testing platform with a considerable number of applications. Covering the complete developmental and reproductive cycle is predicted to reduce the number of false negative results in which chemicals with known toxicity in mammals do not show an effect when using assays with a single end-point^14^. We have tested our system on a range of chemicals of relevance to the agrochemical, pharmaceutical, cosmetics and petrochemical industries, thus demonstrating wide applicability. The compounds tested show toxicity by a wide variety of mechanisms as well as diversity in chemical structure, demonstrating the versatility of this assay.

We hypothesized that the cuticle presented a barrier to chemical uptake and therefore that worms with defects in cuticle structure would show enhanced sensitivity to chemicals due to increased uptake. Some cuticle integrity mutants have already been used to enhance the sensitivity of compound exposures^38, 39^, but there is no consensus on which strain is best. In this report, we have performed a comparative analysis of available strains with defects in the cuticle.

All the mutant strains showed some loss of fitness in terms of developmental timing and brood size. This was most apparent in the *bus-17* and *bus-8* mutants and these were not analysed further as the developmental defects would interfere with the assay for DART. All the remaining cuticle mutants tested showed enhanced sensitivity to a range of chemicals with varying properties, and using a range of solvents, suggesting that the worm cuticle does indeed represent a significant barrier to chemical uptake. The *bus-16* mutant was discounted as it showed sensitivity to sunflower oil, a solvent useful for volatile chemicals^37^ and those with limited solubility in other solvents. Taking all the data into account, the cuticle barrier is best overcome in *bus-5(br19)* which offers optimal sensitivity without problematic loss of fitness. The use of *bus-5* enables much lower concentrations of chemical to cause a robust toxicity outcome, in some cases facilitating detection of adverse outcome that would be missed using WT worms. *C. elegans* is also frequently used in screens to identify drugs causing defined phenotypic changes or to identify genes causing sensitivity or resistance to compounds by targeted reduction of gene expression using genome-wide RNAi^40–45^ Use of *bus-5* will enhance the sensitivity of these screens, increasing the number of hits identified while using reduced amounts of chemical, which can be expensive or limiting in availability.

Overall, we propose that use of *bus-5* could constitute a general replacement of the WT based platform for chemical toxicity screening in these circumstances. However, it will always be important to test chemical effects in more than one genetic background, even if a higher dose is required, to check that the response to a particular chemical is not specific to the mutant background used.

We do not envisage that *C. elegans* would ever completely replace vertebrates in toxicity testing. *C. elegans* could be usefully incorporated into a battery of assays (including other *in vivo* systems like zebrafish and *in vitro* cell-based assays as well as *in silico* modelling) to provide predictive power of a chemical’s toxicity towards humans. Such predictive toxicology would be useful in the elimination of high risk compounds early in product development. Use of *C. elegans* has added value not only in terms of animal replacement but also in the ability to exploit their genetic potential to provide detailed information of the pathways affected by chemicals. This will facilitate rational design of related compounds to minimise the potential for DART without compromising the useful properties of these compounds.

## Methods

### Strains and maintenance

*C. elegans* strains were cultivated as described^46^ and maintained at 20 °C. The strains Bristol N2 and LC144 *[agmo-1(e3016)]* III^29^ were obtained from the *C. elegans* Genetics Center (CGC, University of Minnesota, USA). The following strains were obtained from Jonathan Hodgkin (Dept of Biochemistry, University of Oxford, UK): CB7395 *[bus-5(e2985)]* X, DC19 *[bus-5(br19)]* X, CB6055 *[bus-8(e2698)]* X, CB6177 *[bus-8(e2883)]* X, CB5680 *[bus-16(e2802)]* X, CB5653 *[him-5(e1490)* V; *bus-17(e2800)* X] and CB7431 *[bus-17(br2)]* X. The *him-5* mutation was crossed out of CB5653 to generate the strain AW1452 *[bus-17(e2800)]* X.

### Genotyping primers

Strains were verified by PCR amplification of the relevant locus followed by DNA sequencing using the following primer pairs: XWO1 (5-aaaattaagaaccgcggtgactg) and XWO4 (5-aaaattctgcaaaaattggctcgc) for *bus-17(e2800)*; XWO5 (5-gagaaaagctttggcggcg) and XWO8 (5-atgctcagccaatgcttcaag) for *agmo-1(e3016)*; XWO9 (5-caggtcgatatggtgattcac) and XWO12 (5-ttccacatacatccaacttctag) for *bus-5(br19)*.

### Cuticle sensitivity and permeability assays

Cuticle sensitivity to hypochlorite was tested as described^24, 29^ with modifications. In brief, individual animals (L4 larvae or young gravid adults) were transferred into a 10 μl drop of freshly prepared 2%(v/v) alkaline hypochlorite solution (4 ml 5% sodium hypochlorite, 2.5 ml 4 N NaOH, 3.5 ml dH_2_O) on parafilm and the time taken until first visible rupture was noted.

Cuticle permeability to acridine orange (AO) and 4′,6-diamidino-2-phenylindole (DAPI) was assayed as described^19, 26, 31, 34^ with modifications. L4 larvae were washed from plates with M9 buffer prior to staining with AO or DAPI (5 μg/ml each in M9 buffer) for 15 minutes at room temperature with gentle agitation. Subsequently, worms were washed three times with M9 buffer, followed by fluorescence imaging. For microscopy, worms were mounted onto 3%(w/v) agarose pads, anaesthetised with 10 μl of 1 mM sodium azide and sealed with a coverslip before imaging on a Zeiss Axioplan 2 microscope. Samples were observed with a Zeiss Plan Neofluar 20x/0.50 Ph2 objective, images captured using a Zeiss AxioCam and the software AxioVision 4.8. AO or DAPI accumulation was imaged at 100msec exposure time, except for the strains CB7431 *[bus-17(br2)]* and AW1452 *[bus-17(e2800)]*, where images were taken at 50msec exposure time.

### Plate based toxicity assay

To synchronize worm populations, strains were washed from plates with M9 buffer, residual bacteria were removed by washing worms twice with M9 buffer prior to bleaching gravid adults using 1%(v/v) alkaline hypochlorite solution (2 ml 5% sodium hypochlorite, 1 ml 4 N NaOH, 7 ml dH_2_O) to obtain eggs. Wild type N2 worms dissolved after 10–12 minutes at room temperature, while all mutant strains required 3–5 minutes to dissolve. Hypochlorite was removed by washing the eggs three times with M9 buffer. Eggs were left to hatch overnight at 15 °C to give rise to a population of synchronized L1 larvae. Each well of a 24-well plate containing 500 μl NGM agar was seeded with 20 μl of *Escherichia coli* (OP50) from an overnight culture. The bacterial lawn was allowed to grow at room temperature for two days. For exposure to chemicals dissolved in water, 1%(v/v) DMSO or a mixture containing 1%(v/v) DMSO and 5%(v/v) isopropanol a 30 μl drop of each chemical at the appropriate concentration (or solvent control), sufficient to cover the entire surface of the well, was dried on the agar at room temperature for 30 min in a laminar flow hood. Once the bacterial plates were prepared in this way, five synchronized L1 larvae were picked onto each well. Plates were incubated at 20 °C and an image of each individual well was captured daily using a Leica MZFLIII microscope and Hamamatsu digital camera (Orca-05G) until the bacterial lawn was completely consumed by the worms.

To calculate chemical exposure concentration, the volume of agar was taken into account. For instance, dropping 30 μl of 20 mM boric acid onto 500 μl NGM agar resulted in a final concentration of 1.2 mM boric acid during exposure of L1 larvae. Each chemical concentration was tested in at least three replicate wells.

For exposure to volatile chemicals, e.g. toluene and 2-methoxyethanol, chemicals were mixed with sunflower oil before layering each seeded well with 0.5 ml of chemical-oil mix above the bacterial lawn, after adding the worms. Plates were sealed with parafilm throughout the experiment.

### Brood size determination

L4 larvae (n = 20 animals per strain) were maintained individually under standard conditions. At the onset of egg-laying worms were transferred to a fresh plate daily until egg laying ceased (around 3 days). Progeny from each plate were counted and added together.

### Assessment of developmental fitness

L1 larvae (n = 28 animals per strain) were maintained individually under standard conditions and the number of animals in each developmental stage was noted every 24 hours for three days until egg-laying began.

### Statistical analyses

All statistical analyses were performed using GraphPad Prism. Significance for hypochlorite sensitivity and brood size of individual strains was tested by multiple comparisons using one-way ANOVA. Statistical significance for delay in food depletion during chemical exposure was analysed by comparison of mutant strains to wild type N2 using two-way ANOVA tests.

### Data availability statement

All data generated or analysed during this study are included in this published article (and its Supplementary Information files).

## Electronic supplementary material

Supplementary Figure S1
