# Supplementary material for: An enhanced C. elegans based platform for toxicity assessment
Source: Sci Rep. 2017 Aug 29;7:9839. doi: 10.1038/s41598-017-10454-3 (PMC5575006; doi:10.1038/s41598-017-10454-3)

**An enhanced *C. elegans* based platform for toxicity assessment**

Huajiang Xiong, Catherine Pears* and Alison Woollard*

Department of Biochemistry, University of Oxford, South Parks Road, Oxford OX1 3QU, UK

*co-corresponding authors

**Supplementary Figure S1: Tolerance of sensitised strains to solvents used.** Toxicity assays were set up in 24-well plates as described in the legend to Figure 1 in the presence of a range of concentrations of DMSO or a mixture of DMSO and isopropanol. Images were taken on the day that the untreated strain depleted the food source. From this, the maximum final concentrations of solvent that did not cause developmental effects and therefore used in subsequent experiments were 0.06% DMSO or 0.06 % DMSO/0.3% isopropanol. Scale bar is 1 mm.


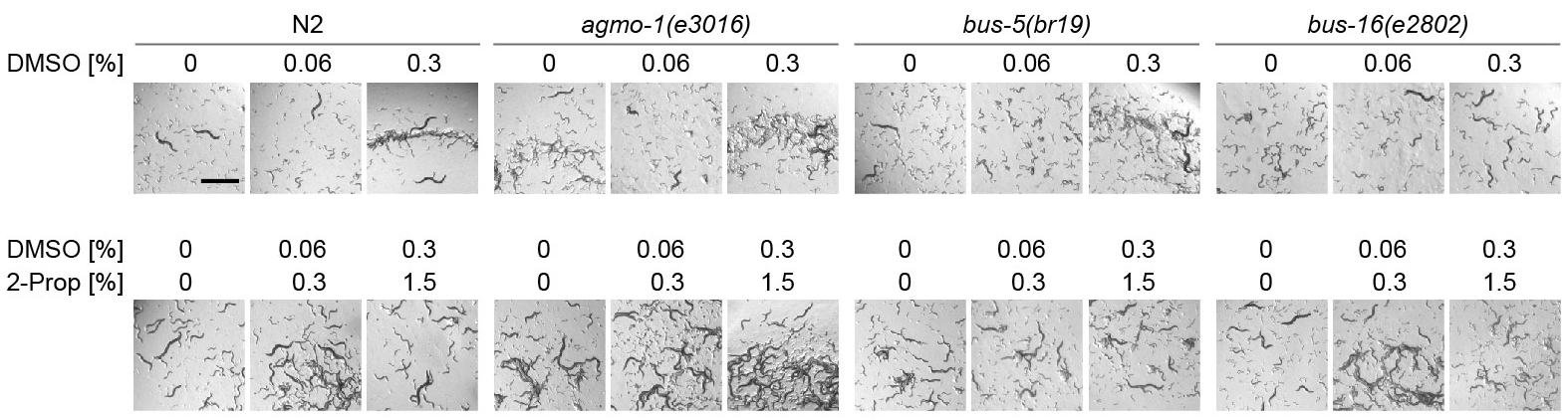

Supplement: Supplementary file 1 — Supplementary Figure S1 [file 41598_2017_10454_MOESM1_ESM.doc]
